# Comparative outcomes of influenza, severe acute respiratory syndrome coronavirus 2, and respiratory syncytial virus community-acquired pneumonia: insights from an Italian retrospective cohort study

**DOI:** 10.1016/j.ijregi.2026.101032

**Published:** 2026-10-04

**Authors:** Margherita S. Silani, Anna Stainer, Massimiliano Russo, Antonio Desai, Sofia Giuriolo, Aniello Fabio Vitale, Vincenzo Alberto Artuso, Angela Tramontano, Francesco Amati, Beatrice Clemente, Alessandra Belati, Francesco Blasi, Michele Bartoletti, Valeria Cento, Stefano Aliberti, Antonio Voza

**Affiliations:** 1 Department of Biomedical Sciences, Humanitas University, Pieve Emanuele, Italy; 2 Department of Medical Biotechnologies, University of Siena, Siena, Italy; 3 Respiratory Unit, IRCCS Humanitas Research Hospital, Milan, Italy; 4 Emergency Department, IRCCS Humanitas Research Hospital, Milan, Italy; 5 Microbiology, IRCCS Humanitas Research Hospital, Milan, Italy; 6 Department of Pathophysiology and Transplantation, University of Milan, Milan, Italy; 7 Respiratory and Cystic Fibrosis Unit, Internal Medicine Department, Fondazione IRCCS Ca’ Granda Ospedale Maggiore Policlinico, Milan, Italy; 8 Infectious Disease Unit, IRCCS Humanitas Research Hospital, Rozzano, Italy

**Keywords:** Community-acquired pneumonia, CAP, Viral pneumonia, Influenza, RSV, SARS-CoV-2

## Abstract

Community-acquired pneumonia (CAP) is a leading cause of morbidity and mortality worldwide. Viral pathogens, particularly influenza, respiratory syncytial virus, and severe acute respiratory syndrome coronavirus 2 (SARS-CoV-2), are increasingly recognized as key contributors to CAP in hospitalized adults. This single-center, retrospective cohort study examined and compared the clinical characteristics and outcomes of adults hospitalized with viral CAP due to influenza, SARS-CoV-2, or respiratory syncytial virus between January 2022 and December 2023 in Milan, Italy. A total of 165 patients with laboratory-confirmed single-virus infection and clinically and radiologically confirmed pneumonia were included. Patients with SARS-CoV-2 pneumonia presented with the highest in-hospital mortality (20.9%) and 12-month all-cause mortality (51.9%). Patients with influenza, although younger, showed increased rates of cardiovascular events and intensive care unit admissions. Bacterial coinfections were most common in influenza cases. Immunocompromised status was the second most frequent comorbidity (37.6%) after arterial hypertension. These findings underscore significant differences in clinical trajectory and long-term outcomes among major viral CAP etiologies and support the need for pathogen-specific management strategies.

## Highlights

• Viral pneumonia affects predominantly elderly patients with multiple comorbidities.
• Influenza had higher rates of bacterial coinfection and cardiovascular events.
• Pneumonia severity had an increasing trend from respiratory syncytial virus to influenza to severe acute respiratory syndrome coronavirus 2.
• Despite highest vaccination coverage, severe acute respiratory syndrome coronavirus 2 led to highest mortality outcomes.

## Introduction

Community-acquired pneumonia (CAP) is a significant global health challenge, accounting for an estimated 2 million deaths annually [1]. Viral pathogens are increasingly recognized in up to 26% of adult CAP hospitalizations, with influenza and respiratory syncytial virus (RSV) historically being predominant in the pre-pandemic era [2]. Since late 2019, severe acute respiratory syndrome coronavirus 2 (SARS-CoV-2) has emerged as a major cause of viral pneumonia, often surpassing influenza and RSV in severity and mortality [[3], [4], [5]]. Recent comparative studies evaluating the clinical outcomes of these viral etiologies have produced mixed results, particularly when considering the SARS-CoV-2 Omicron variant, updated vaccine coverage, and the recent introduction of RSV vaccines [6,7]. There remains a pressing need for robust data comparing these pathogens to guide future public health and vaccination strategies. This study aimed to evaluate and compare the clinical features, disease severity, and short- and long-term outcomes of hospitalized adults with CAP due to influenza, RSV, or SARS-CoV-2.

## Methods

A retrospective, observational cohort study was conducted at IRCCS Humanitas Research Hospital in Rozzano, Milan, Italy, involving consecutive adults (aged ≥18 years) hospitalized with confirmed viral CAP between January 2022 and December 2023. CAP was defined as an acute illness acquired outside the hospital and characterized by the presence of a new pulmonary infiltrate on chest radiograph or computed tomography, in addition to at least two of the following signs or symptoms: new-onset cough with or without sputum production, pleuritic chest pain, dyspnea, fever or hypothermia, abnormal breath sounds on auscultation, and leukocytosis. Following the hospital’s standard operating procedure, all patients with a suspected lower respiratory tract infection during the study period underwent reverse-transcription polymerase chain reaction or antigen testing for influenza, RSV, and SARS-CoV-2 on a respiratory sample (e.g. nasopharyngeal swab, sputum, bronchial aspirate, or bronchoalveolar lavage) at hospital admission; eligible patients were those who tested positive for one of these viruses and were diagnosed with CAP. Patients with viral coinfections were excluded. Ethical approval was obtained from the local institutional review board (ethics committee: Comitato Etico Territoriale Lombardia 5; Institution: IRCCS Humanitas Rozzano; approval number 31/23). The primary outcome was in-hospital all-cause mortality. Secondary outcomes included all-cause mortality at 1, 6, and 12 months. Local standard operating procedures for access or transfer to the intensive care unit and infection control had been in place since before the study and were stable during the entire study period. Patient follow-up was obtained through institutional and regional electronic medical records. Comparative statistical analyses included Fisher’s exact test or chi-square test for categorical variables and one-way analysis of variance, Student’s independent *t*-test, Kruskal–Wallis test, or Mann–Whitney U test for continuous variables. The association between viral etiology and in-hospital mortality was assessed using Firth’s penalized logistic regression adjusted for Pneumonia Severity Index (PSI) class. All-cause mortality in the complete population and by viral etiology up to 12 months was described with Kaplan–Meier curves and compared using the log-rank test. Patients who died during hospitalization were excluded, and patients lost to follow-up were censored at the last time point known to be alive. Significance was defined as *P* < 0.05. Missing data exceeding 10% are indicated for the involved variables. Given that the pattern of missingness was judged unlikely to be missing completely at random, multiple imputation was not considered appropriate; analyses were, therefore, conducted on an available-case basis.

## Results

Among 165 patients (61.2% male, median [interquartile range] age: 77 [63-86] years), 17 had RSV, 57 had influenza, and 91 had SARS-CoV-2 pneumonia (Table 1). Patients with influenza were significantly younger than patients with RSV and SARS-CoV-2. Vaccination coverage was suboptimal for influenza (47.5%) and pneumococcus (18.4%), whereas SARS-CoV-2 vaccination reached 87.8%, although a high number of missing cases must be noted. Immunosuppression was the second most common comorbidity (37.6%) and biological therapy was reported exclusively in patients with SARS-CoV-2 (8.8% vs 0% in RSV and influenza, *P* = 0.036). Among respiratory comorbidities, asthma was more frequent in influenza cases (12.3% vs 5.9% in RSV and 2.2% in SARS-CoV-2, *P* = 0.038). Vital parameters, laboratory panel, radiology and symptoms at admission are depicted in Supplementary Table 1. Pneumonia severity was significantly highest in the SARS-CoV-2 group, as measured by CURB-65 and PSI scores. At admission, cardiovascular events (21.1% vs 0% in RSV and 5.5% in SARS-CoV-2, *P* = 0.005) and intensive care unit admissions (12.3% vs 0% in both other groups, *P* < 0.001) were more frequent in influenza cases. Bacterial coinfections occurred most frequently among patients with influenza (14%, *P* = 0.003). Overall, 129 (78.2%) patients started empirical antibacterial treatment within the first 24 hours from admission, of which 40% consisted of combination therapy (Supplementary Table S2). The most frequent antimicrobial agents were ceftriaxone (71, 43%), piperacillin/tazobactam (46, 27.9%), and remdesivir (42, 25.5%). Concomitant steroid use was high in influenza and SARS-CoV-2 pneumonia (56.4 and 51.7%, respectively). In-hospital mortality was highest among SARS-CoV-2 cases (20.9%), but after adjustment for PSI class, viral etiology was not independently associated with in-hospital mortality (overall *P* = 0.40), whereas a higher PSI class was (adjusted odds ratio 3.07 per class increase, 95% confidence interval 1.46-6.99, *P* = 0.002) (Table 2, Supplementary Table S3). Among 141 patients discharged alive, 10 died during follow-up: the Kaplan–Meier estimated 12-month survival was 87.8% (95% confidence interval 80.6-95.5), with no significant difference between RSV (94.1%), influenza (94.1%), and SARS-CoV-2 (81.3%) (log-rank *P* = 0.12) (Supplementary Figures S1 and S2).

**Table 1:** Demographics, comorbidities, disease severity, and characteristics of the study population according to the three study groups.

Variable	Respiratory syncytial virus (n = 17)	Influenza (n = 57)	SARS-CoV-2 (n = 91)	*P*-value
**Demographics**				
Age, median (IQR) years	78 (66-88)	70 (60.5-84.5)	79 (67-86)	0.100
Age >65 years, N (%)	14 (82.4)	34 (59.6)	73 (80.2)	**0.015^a^**
Male sex, N (%)	8 (47.1)	30 (52.6)	63 (69.23)	0.059
Either active or previous smoker, N (%)^c^	14 (82.3)	26 (52)	34 (75.6)	**0.016^b^**

*^a^P*-values: RSV vs influenza = 0.085, RSV vs SARS-CoV-2 = 1.000, influenza vs SARS-CoV-2= 0.007. ^b^*P*-values: RSV vs influenza = 0.028, RSV vs SARS-CoV-2 = 0.739, influenza vs SARS-CoV-2= 0.017. ^c^Missing 53.				

**Vaccination status**				
Flu vaccination in the previous 12 months, N (%)^d^	4 (66.7)	7 (41.2)	8 (47.1)	0.631
Any pneumococcal vaccination, N (%)^e^	3 (50)	2 (11.1)	2 (14.3)	0.152
SARS-CoV-2 vaccination, N (%)^f^	6 (100)	38 (90.5)	35 (83.3)	0.512
SARS-CoV-2 vaccination, median (IQR) doses^¶¶¶^	3 (3-4)	3 (3-3)	3 (2-3)	0.405

^d^ Missing 125. ^e^ Missing 127. ^f^ Missing 75.				

Essential hypertension, N (%)	11 (64.7)	33 (57.9)	62 (68.1)	0.449
Immunocompromised, N (%)	5 (29.4)	23 (40.4)	34 (37.4)	0.715
COPD, N (%)	5 (29.4)	10 (17.5)	25 (27.5)	0.340
Diabetes, N (%)	6 (35.3)	9 (15.8)	23 (25.3)	0.178
Atrial fibrillation, N (%)	4 (23.5)	13 (22.8)	21 (23.1)	1.000
Coronary artery disease, N (%)	3 (17.6)	10 (17.5)	21 (23.1)	0.750
Congestive heart failure, N (%)	2 (11.8)	9 (15.8)	17 (18.7)	0.867
Previous cerebrovascular event, N (%)	2 (11.8)	8 (14.0)	17 (18.7)	0.743
Chronic renal failure, N (%)	1 (5.9)	5 (8.9)	17 (18.7)	0.204
Hematologic cancer, N (%)	2 (11.8)	5 (8.8)	17 (18.7)	0.265
Active malignancy or malignancy within 1 year of CAP, N (%)	1 (5.9)	9 (15.8)	17 (18.7)	0.504
Neurologic or mental illness, N (%)	2 (11.8)	16 (28.1)	15 (16.5)	0.175
Chemotherapy last 3 months, N (%)	1 (5.9)	7 (12.3)	14 (15.4)	0.631
Previous acute myocardial infarction, N (%)	4 (23.5)	6 (10.5)	11 (12.1)	0.374
Prior admissions for CAP within 1 year, N (%)	1 (5.9)	4 (7.0)	10 (11.0)	0.784
Other solid tumor with neutropenia or chemotherapy, N (%)	0 (0.0)	4 (7.0)	8 (8.8)	0.621
Bronchiectasis, N (%)	3 (17.6)	6 (10.5)	8 (8.8)	0.485
Other immunocompromised, N (%)	1 (5.8)	3 (5.2)	8 (8.9)	0.820
Biological drug, N (%)	0 (0.0)	0 (0.0)	8 (8.7)	**0.036^g^**
Hematopoietic cell transplantation, N (%)	0 (0.0)	2 (3.5)	5 (5.4)	0.865
Neutropenia, N (%)	0 (0.0)	2 (3.5)	5 (5.5)	0.865
Suspicion of aspiration, N (%)	0 (0.0)	4 (7.0)	7 (7.7)	0.731
Prednisone 10 mg/day ≥3 months, N (%)	3 (17.6)	11 (19.3)	2 (2.2)	**<0.001^h^**
Prednisone 20 mg/day ≥14 days or cumulative dose >600 mg, N (%)	3 (17.6)	7 (12.3)	2 (2.1)	**0.009^i^**
Asthma, N (%)	1 (5.9)	7 (12.3)	2 (2.2)	**0.038^j^**
Solid organ transplantation, N (%)	0 (0.0)	2 (3.5)	1 (1.1)	0.682
Lung cancer with neutropenia or chemotherapy, N (%)	0 (0.0)	1 (1.8)	3 (3.2)	1.000
AIDS, N (%)	0 (0.0)	1 (1.8)	0 (0.0)	0.448
Aplastic anemia, N (%)	0 (0.0)	0 (0.0)	1 (1.1)	1.000
Asplenia, N (%)	0 (0.0)	0 (0.0)	0 (0.0)	-

^g^*P*-values: RSV vs influenza = NA, RSV vs SARS-CoV-2 = 0.204, influenza vs SARS-CoV-2= 0.024. ^h^*P*-values: RSV vs influenza = 1.000, RSV vs SARS-CoV-2 = 0.027, influenza vs SARS-CoV-2 ≤0.001. ^i^*P*-values: RSV vs influenza = 0.687, RSV vs SARS-CoV-2 = 0.027, influenza vs SARS-CoV-2= 0.028. ^j^*P*-values: RSV vs influenza = 0.673, RSV vs SARS-CoV-2 = 0.405, influenza vs SARS-CoV-2 = 0.028.				

**Severity at admission**				
Hypoxemia with necessity of mechanical ventilation, N (%)	1 (5.9)	7 (12.3)	8 (8.9)	0.735
Vasopressors, N (%)	0	3 (5.2)	0	0.105
ARDS, N (%)	0	5 (8.8)	10 (11)	0.523
ARF within the first 24-48 hours, N (%)	10 (58.8)	19 (33.3)	44 (48.4)	0.093
ICU at admission, N (%)	0	7 (12.3)	0	**<0.001^k^**
Any cardiovascular event (at least one), N (%)	0	12 (21.1)	5 (5.5)	**0.005^l^**
AMI on admission, N (%)	0	0	1 (1.1)	1.000
Acute heart failure, N (%)	0	3 (5.3)	2 (2.2)	0.639
New arrhythmia, N (%)	0	7 (12.3)	1 (1.1)	**0.008^m^**
Endocarditis, N (%)	0	1 (1.8)	0	0.448
Cerebrovascular event, N (%)	0	1 (1.8)	2 (2.2)	1.000
sCAP, N (%)	0	12 (21.1)	21 (23.1)	0.062
PSI score, median (IQR)	87 (69.5-96)	102 (80.5-120.5)	119 (104-136)	**<0.001^n^**
PSI class, median (IQR)	3 (2-4)	4 (3-4)	4 (4-5)	**<0.001^o^**
CURB-65 score, median (IQR)	1 (1-1)	1 (1-2)	2 (1-2)	**<0.001^p^**

^k^*P*-values: RSV vs influenza = 0.129, RSV vs SARS-CoV-2 = NA, influenza vs SARS-CoV-2 ≤0.001. ^l^*P*-values: RSV vs influenza = 0.039, RSV vs SARS-CoV-2 = 1.000, Influenza vs SARS-CoV-2 = 0.004. ^m^*P*-values: RSV vs influenza = 0.129, RSV vs SARS-CoV-2 = 1.000, influenza vs SARS-CoV-2 = 0.005. ^n^*P*-values: RSV vs influenza = 0.07, RSV vs SARS-CoV-2 ≤0.001, influenza vs SARS-CoV-2 ≤0.001. ^o^*P*-values: RSV vs influenza = 0.010, RSV vs SARS-CoV-2 ≤0.001, influenza vs SARS-CoV-2 ≤0.001. ^p^*P*-values: RSV vs influenza = 0.027, RSV vs SARS-CoV-2 ≤0.001, influenza vs SARS-CoV-2 = 0.057.				

**Respiratory support on admission**				
CPAP T0, N (%)	0	1 (1.8)	5 (5.5)	0.565
NIV T0, N (%)	1 (5.9)	0	5 (5.5)	0.146
O2 T0, N (%)	9 (52.9)	12 (21.1)	27 (29.7)	**0.044^q^**
ETI T0, N (%)	0	3 (5.3)	0	0.105
HFNC T0, N (%)	0	3 (5.3)	1 (1.1)	0.325
^q^*P*-values: RSV vs influenza = 0.010, RSV vs SARS-CoV-2 = 0.062, influenza vs SARS-CoV-2 = 0.247.				


Abbreviations: AIDS, acquired immunodeficiency syndrome; AMI, acute myocardial infarction; ARDS, acute respiratory distress syndrome; ARF, acute respiratory failure; CAP, community-acquired pneumonia; COPD, chronic obstructive pulmonary disease; CPAP, continuous positive airway pressure; CVE, cardiovascular event; ETI, endotracheal intubation; HFNC, high-flow nasal cannula; ICU, intensive care unit; IQR, interquartile range; NIV, noninvasive ventilation; O₂, oxygen; PSI, Pneumonia Severity Index; RSV, respiratory syncytial virus; SARS-CoV-2, severe acute respiratory syndrome coronavirus 2; sCAP, severe community-acquired pneumonia.

**Table 2:** Disease outcomes of the study population according to the three study groups.

	Respiratory syncytial virus (n = 17)	Influenza (n = 57)	SARS-CoV-2 (n = 91)	*P-*value
**Outcomes during hospitalization**				
ICU transfer, N (%)	1 (5.9)	8 (14)	4 (4.4)	0.094
ETI, N (%)	1 (5.9)	7 (12.3)	4 (4.4)	0.162
Bilevel, N (%)	0	1 (1.8)	4 (4.4)	0.798
CPAP, N (%)	0	3 (5.3)	8 (8.8)	0.478
HFNC, N (%)	0	9 (15.8)	11 (12.1)	0.214
Respiratory ICU transfer, N (%)	1 (5.9)	6 (10.5)	3 (3.3)	0.187
Vasopressors, N (%)	0	3 (5.3)	2 (2.2)	0.639
ARDS, N (%)	0	4 (7.0)	4 (4.4)	0.563
ARF, N (%)	2 (11.8)	4 (7.0)	5 (5.5)	0.535
Any cardiovascular event (at least one), N (%)	1 (5.9)	10 (17.5)	7 (7.7)	0.142
Acute heart failure, N (%)	0	3 (5.3)	3 (3.3)	0.833
New arrhythmia, N (%)	1 (5.9)	7 (12.3)	2 (2.2)	**0.038^a^**
Cerebrovascular, N (%)	0	0	2 (2.2)	0.617
Hospital stay, median (IQR) days	13 (8.5-16)	12 (8-20)	11 (7-17)	0.392
In-hospital mortality, N (%)	0	5 (8.8)	19 (20.9)	**0.022^b^**

**^a^***P*-values: RSV vs influenza = 0.673, RSV vs SARS-CoV-2 = 0.396, influenza vs SARS-CoV-2= 0.028. ^b^*P*-values: RSV vs influenza = 0.583, RSV vs SARS-CoV-2 = 0.038, influenza vs SARS-CoV-2 = 0.052.				

**Outcomes after discharge**				
Hospital admission after 1 month, N (%)^c^	1 (5.9)	4 (13.3)	7 (12.7)	0.837
Hospital admission after 6 months, N (%)^d^	2 (11.8)	9 (36)	13 (31)	0.137
Hospital admission after 12 months, N (%)^e^	6 (35.3)	10 (45.5)	15 (37.5)	0.772
New CVE after 1 month, N (%)^f^	2 (11.8)	10 (26.3)	7 (10.9)	0.138
New CVE after 6 months, N (%)^g^	3 (17.6)	10 (37)	8 (16)	0.109
New CVE after 12 months, N (%)^h^	3 (17.6)	10 (37)	8 (17.4)	0.163
All-cause mortality after 1 month, N (%)	0	5 (8.8)	22 (24.2)	**0.007^i^**
All-cause mortality after 6 months, N (%)^j^	1 (5.9)	5 (22.7)	26 (48.1)	**0.002^k^**
All-cause mortality after 12 months, N (%)^l^	1 (5.9)	6 (27.3)	27 (51.9)	**0.001^m^**

^c^ Missing 63. ^d^ Missing 81. ^e^ Missing 86. ^f^ Missing 46. ^g^ Missing 71. ^h^ Missing 75. ^i^ P-values: RSV vs influenza = 0.583, RSV vs SARS-CoV-2 = 0.021, influenza vs SARS-CoV-2 = 0.018. ^j^ Missing 72. ^k^ P-values: RSV vs influenza = 0.206, RSV vs SARS-CoV-2 = 0.002, influenza vs SARS-CoV-2 = 0.041. ^l^ Missing 74. ^m^ P-values: RSV vs influenza = 0.113, RSV vs SARS-CoV-2 ≤0.001, influenza vs SARS-CoV-2 = 0.051.				


Abbreviations: ARDS, acute respiratory distress syndrome; ARF, acute respiratory failure; CAP, community-acquired pneumonia; COVID-19, coronavirus disease 2019; CPAP, continuous positive airway pressure; CVE, cardiovascular event; ETI, endotracheal intubation; HFNC, high-flow nasal cannula; ICU, intensive care unit; IQR, interquartile range; NIV, noninvasive ventilation; RSV, respiratory syncytial virus; SARS-CoV-2, severe acute respiratory syndrome coronavirus 2.

## Discussion

This study highlights substantial differences in presentation, management, and outcomes among adults hospitalized with CAP due to RSV, influenza, or SARS-CoV-2. Despite high vaccination coverage, SARS-CoV-2 was associated with the highest mortality, supporting the notion that coronavirus disease 2019 remains a significant health burden even in the Omicron era and suggesting the necessity to better understand the long-term efficacy of vaccination and eventually implementing boosters in specific patient categories [8]. Influenza cases demonstrated increased cardiovascular complications, consistent with previous findings suggesting heightened thrombo-inflammatory risk. Therefore, although patients with influenza showed better mortality outcomes, these data underscore the necessity of higher vaccine coverage in fragile populations to prevent adverse events. The observed bacterial coinfection rate in patients with influenza further supports the need for timely antimicrobial strategies in this group. Notably, RSV-related CAP had the lowest severity and mortality, aligning with recent data from adult-only cohorts, although differences in comorbidity burden and health care–seeking behavior warrant further investigation, and the low case number limits the reliability of the data [[5], [6], [7]]. These results should be interpreted considering the changed epidemiology of viral respiratory infections during and after the coronavirus disease 2019 pandemic: compared with the pre-pandemic era, in the 2022/2023 season, RSV and influenza peaked earlier and lasted longer in the northern hemisphere [9]. More recently, SARS-CoV-2, influenza, and RSV have co-circulated in Italy among elderly, comorbid patients hospitalized with severe acute respiratory infections, supporting the continued clinical burden of all three viruses observed in our cohort [10]. Study limitations include its retrospective, monocentric design, leading to missing data in some relevant variables, such as vaccine status, and a limited sample size. Moreover, long-term mortality was analyzed descriptively because of the small number of events and incomplete follow-up. Nonetheless, this study uniquely offers long-term follow-up across three major viral CAP etiologies within a single, standardized health care setting.

## Conclusions

Our findings emphasize the heterogeneity of viral CAP in hospitalized adults. Although SARS-CoV-2 is still associated with high long-term mortality, influenza and RSV also pose significant clinical challenges. These data reinforce the need for pathogen-specific prevention and management strategies and suggest that long-term outcomes should be considered in the development of vaccination and therapeutic policies.

## Funding

This research did not receive any specific grant from funding agencies in the public, commercial, or non-profit sectors.

## Ethical approval

Verbal informed consent to take part in the study and publish the article was obtained from all participants or their legal representatives. Due to the retrospective nature of the study, no informed consent was necessary. The privacy rights of the participants have been observed.

This study was performed in compliance with the relevant laws, regulatory frameworks, and guidelines where the research took place. This study was approved by the Comitato Etico Territoriale Lombardia 5 (approval no. 31/23).

## Data availability

The data that support the findings of this study are available from the corresponding author, Stefano Aliberti, upon reasonable request.

## CRediT authorship contribution statement

**Margherita S. Silani:** Data curation, Formal analysis, Methodology, Writing – original draft, Writing – review & editing. **Anna Stainer:** Conceptualization, Data curation, Formal analysis, Methodology, Project administration, Software, Writing – review & editing. **Massimiliano Russo:** Data curation, Writing – review & editing. **Antonio Desai:** Data curation, Writing – review & editing. **Sofia Giuriolo:** Data curation, Writing – review & editing. **Aniello Fabio Vitale:** Data curation, Writing – review & editing. **Vincenzo Alberto Artuso:** Data curation, Writing – review & editing. **Angela Tramontano:** Data curation, Writing – review & editing. **Francesco Amati:** Data curation, Writing – review & editing. **Beatrice Clemente:** Data curation, Writing – review & editing. **Alessandra Belati:** Data curation, Writing – review & editing. **Francesco Blasi:** Conceptualization, Writing – review & editing. **Michele Bartoletti:** Writing – review & editing, Project administration. **Valeria Cento:** Writing – review & editing, Project administration. **Stefano Aliberti:** Conceptualization, Data curation, Methodology, Project administration, Supervision, Writing – original draft, Writing – review & editing. **Antonio Voza:** Conceptualization, Data curation, Methodology, Supervision, Writing – review & editing.

## Declaration of competing interest

MSS, AS, MR, AD, SG, AFV, VAA, AT, FA, BC, and AB have no conflicts of interest or competing interests to report.

FB reports grants from AstraZeneca, Chiesi, and INSMED; personal fees from Menarini; and payments for lectures and advisory boards from AstraZeneca, Chiesi, Boehringer Ingelheim, Menarini, Novartis, Om Pharma, Pfizer, Sanofi, Vertex, and Zambon.

MB reports grants from Biomerieux, Tillots, and MSD and consulting fees from Gilead, Pfizer, Advanz, Menarini, Biomerieus, Infectopharm, and MSD.

VC reports grants from Sansure; consulting fees from Zambon; payments for honoraria or lectures from Cepheid, SD Biosensor, Pfizer, and MundiPharma; and support for meetings or travel from Abbott, Elitech Group, and SD Biosensor.

SA reports consulting fees from GSK, INSMED, Zambon, AstraZeneca, Menarini, CSL Behring, Pfizer, Moderna, Boehringer, Chiesi, MSD, Vertex, Brahms, Physioassist, AN2 Therapeutics, GSK, and Verona; payments for lectures from GSK, Menarini, INSMED, Boehringer, Zambon, and Vertex; and participation on boards for INSMED, AstraZeneca, MSD, and Verona.

AV reports grants from EU, payments for lectures from Abbott and Angelini, payments for expert testimony from AstraZeneca, and support for attending meetings or travel from GSK.

## Appendix Supplementary materials

Image, application 1

Image, application 2
