# Supplementary material for: Comparative outcomes of influenza, severe acute respiratory syndrome coronavirus 2, and respiratory syncytial virus community-acquired pneumonia: insights from an Italian retrospective cohort study
Source: IJID Reg. 2026 Oct 4;21:101032. doi: 10.1016/j.ijregi.2026.101032 (PMC13757497; doi:10.1016/j.ijregi.2026.101032)
Supplement: Supplementary file 1 [file mmc2.pdf]

Supplementary material to:

Comparative Outcomes of Influenza, SARS-CoV-2, and Respiratory Syncytial Virus Community-Acquired Pneumonia: Insights from an Italian Retrospective Cohort Study

Table of content

**Supplementary Table S1**	**Page nr. 3**
**Supplementary Table S2**	**Page nr. 5**
**Supplementary Table S3**	**Page nr. 6**
**Supplementary Figure S1**	**Page nr. 7**
**Supplementary Figure S2**	**Page nr. 8**

Variable	Respiratory Syncytial Virus (n=17)	Influenza (n=57)	SARS-CoV-2 (n=91)	P-value
**Vital Parameters at admission**				
Systolic Blood Pressure, median (IQR) mmHg	130 (105-172)	125 (110-143)	130 (110-146)	0.648
Diastolic Blood Pressure, median (IQR) mmHg	75 (68.5- 82)	74 (65.5-84)	74 (62-80)	0.778
Heart Rate, median (IQR) beats/min	85 (82-104.5)	96 (82-111.5)	87.5 (77-100)	0.064
Respiratory Rate, median (IQR) /min	18 (18-22)	18 (18-20.5)	18 (18-23)	0.986
Oxygen saturation, median (IQR) %	95 (94-96)	95 (92-97)	95 (90-97)	0.845
Temperature, median (IQR) °C	37 (36-38.1)	37 (36.1-38)	36.8 (36-38)	0.951
**Laboratory Tests at admission**				
White Blood Cells, median (IQR) /mm³	11060 (7130-15070)	7685 (6175-11680)	7700 (5735-11175)	0.101
Hemoglobin, median (IQR) g/dL	12.8 (11.9-13.5)	12.3 (10.8-14.4)	12.5 (11.1-13.8)	0.747
Platelet Count, median (IQR) ×10³/mm³	230 (167.8-252)	199.5 (136.3-295.1)	174 (124.5-237)	0.107
Urea, median (IQR) mg/dL	42.5 (35.5-83.8)	51 (37-67)	52 (35-83)	0.870
Creatinine, median (IQR) mg/dL	1.02 (0.8-1.45)	0.98 (0.7-1.47)	1.11 (0.85-1.54)	0.454
AST, median (IQR) U/L	25 (20-35.5)	35.5 (27.3-48.8)	28 (22-47)	0.061
ALT, median (IQR) U/L	21 (15-25.5)	24.5 (16-38)	20 (14-35)	0.323
Total Bilirubin, median (IQR) mg/dL	0.6 (0.5-0.9)	0.7 (0.5-1)	0.7 (0.5-0.9)	0.756
Glucose, median (IQR) mg/dL	135 (107.5-171)	116 (102.8- 154)	120 (102- 160.8)	0.390
Troponin, median (IQR) ng/L	19.4 (8.6-32.1)	24.6 (13.1-43.9)	23.8 (10.1-81.5)	0.642
CRP, median (IQR) mg/dL	14.7 (2.8-17.8)	10.8 (4.4-17)	11.4 (4.6-16.5)	0.997
PCT, median (IQR) ng/mL	0.36 (0.12-2.93)	0.36 (0.14-3.27)	0.36 (0.11-1.12)	0.806

**Radiology at presentation**				
Multilobar infiltrate, N (%)	13 (76.4)	43 (75.4)	63 (69.2)	0.716
Interstitial pneumonia, N (%)	1 (5.9)	23 (40.4)	61 (67)	<0.001*
Pleural effusion, N (%)	2 (11.8)	11 (19.3)	34 (37.4)	0.018**
* p-values: RSV vs. Influenza = 0.008, RSV vs. SARS-CoV2 = <0.001, Influenza vs. SARS-CoV-2= 0.001<br>** p-values: RSV vs. Influenza = 0.719, RSV vs. SARS-CoV2 =0.040, Influenza vs. SARS-CoV-2= 0.020				
**Symptoms at admission**				
Cough, N (%)	7 (41.2)	16 (28.1)	28 (30.8)	0.603
Sputum, N (%)	1 (5.9)	8 (14)	9 (9.9)	0.659
Dyspnea, N (%)	11 (64.7)	37 (64.9)	54 (59.3)	0.818
Fever, N (%)	8 (47.1)	33 (57.9)	51 (56)	0.742
Hemoptysis, N (%)	1 (5.9)	2 (3.5)	1 (1.1)	0.235
Asthenia, N (%)	6 (35.3)	34 (59.6)	29 (31.9)	0.003*
Night Sweats, N (%)	0 (0)	1 (1.8)	0 (0)	0.448
Weight Loss, N (%)	0 (0.0)	6 (10.5)	4 (4.4)	0.245
Neurological Symptoms, N (%)	2 (11.8)	5 (8.8)	18 (19.8)	0.203
Gastrointestinal Symptoms, N (%)	0 (0.0)	4 (7)	5 (5.5)	0.787
* p-values: RSV vs. Influenza = 0.077, RSV vs. SARS-CoV2 = 0.782, Influenza vs. SARS-CoV-2= <0.001				

Supplementary Table S1. Vital parameters, laboratory panel, radiology and symptoms at admission in viral pneumonia.

Abbreviations: AST, aspartate aminotransferase; ALT, alanine aminotransferase; CRP, C-reactive protein; PCT, procalcitonin; IQR, interquartile range.

	Overall (N = 165)	RSV (N = 17)	Influenza (N = 57)	SARS-CoV-2 (N = 91)	p-value	Missing, n
**Empirical antibacterial therapy**						
Any antibacterial agent, N (%)	129 (78.2)	15 (88.2)	49 (86.0)	65 (71.4)	0.073	0
Monotherapy, N (%)	63 (38.2)	5 (29.4)	21 (36.8)	37 (40.7)	0.691	0
Combination therapy (≥2 agents) , N (%)	66 (40.0)	10 (58.8)	28 (49.1)	28 (30.8)	0.020	0
**Antibacterial agents**						
Ceftriaxone, N (%)	71 (43.0)	9 (52.9)	24 (42.1)	38 (41.8)	0.699	0
Piperacillin/tazobactam, N (%)	46 (27.9)	3 (17.6)	18 (31.6)	25 (27.5)	0.565	0
Azithromycin, N (%)	43 (26.1)	8 (47.1)	16 (28.1)	19 (20.9)	0.073	0
Levofloxacin, N (%)	19 (11.5)	1 (5.9)	13 (22.8)	5 (5.5)	0.005	0
Vancomycin, N (%)	5 (3.0)	1 (5.9)	2 (3.5)	2 (2.2)	0.391	0
Meropenem, N (%)	4 (2.4)	0 (0.0)	4 (7.0)	0 (0.0)	0.024	0
Amoxicillin/clavulanic acid, N (%)	3 (1.8)	0 (0.0)	1 (1.8)	2 (2.2)	1.000	0
Linezolid, N (%)	3 (1.8)	0 (0.0)	2 (3.5)	1 (1.1)	0.682	0
Fosfomycin, N (%)	2 (1.2)	1 (5.9)	1 (1.8)	0 (0.0)	0.082	0
Trimethoprim/sulfamethoxazole, N (%)	2 (1.2)	0 (0.0)	0 (0.0)	2 (2.2)	0.617	0
Cefiderocol, N (%)	1 (0.6)	0 (0.0)	1 (1.8)	0 (0.0)	0.448	0
Ceftazidime, N (%)	1 (0.6)	1 (5.9)	0 (0.0)	0 (0.0)	0.103	0
Ciprofloxacin, N (%)	1 (0.6)	1 (5.9)	0 (0.0)	0 (0.0)	0.103	0
**Antiviral agents**						
Remdesivir, N (%)	42 (25.5)	0 (0.0)	0 (0.0)	42 (46.2)	<0.001	0
Oseltamivir, N (%)	38 (23.0)	0 (0.0)	38 (66.7)	0 (0.0)	<0.001	0
Nirmatrelvir/ritonavir, N (%)	3 (1.8)	0 (0.0)	0 (0.0)	3 (3.3)	0.485	0
Acyclovir, N (%)	1 (0.6)	0 (0.0)	1 (1.8)	0 (0.0)	0.448	0
**Antifungal agents**						
Amphotericin B, N (%)	1 (0.6)	0 (0.0)	1 (1.8)	0 (0.0)	0.448	0
Anidulafungin, N (%)	1 (0.6)	0 (0.0)	1 (1.8)	0 (0.0)	0.448	0
Voriconazole, N (%)	1 (0.6)	0 (0.0)	0 (0.0)	1 (1.1)	1.000	0

Supplementary Table S2. Antimicrobial therapy stated in the first 24 hours from admission to hospital in the study population with diagnosis of viral pneumonia.

	aOR	95% CI	p-value
Viral etiology (overall)			0.40
RSV vs SARS-CoV-2	0.31	0.002–2.95	0.37
Influenza vs SARS-CoV-2	0.55	0.18–1.51	0.26
RSV vs Influenza	0.57	0.004–6.14	0.69
PSI class (per 1-class increase)	3.07	1.46–6.99	**0.002**

Supplementary Table S3. Multivariable logistic regression for in-hospital mortality according to viral etiology, adjusted for PSI class (n = 165). The overall p-value for viral etiology was obtained with a penalized likelihood-ratio test ($\chi^2 = 1.82$, 2 df). aOR: adjusted odds ratio; CI: confidence interval; PSI: Pneumonia Severity Index; RSV: respiratory syncytial virus.

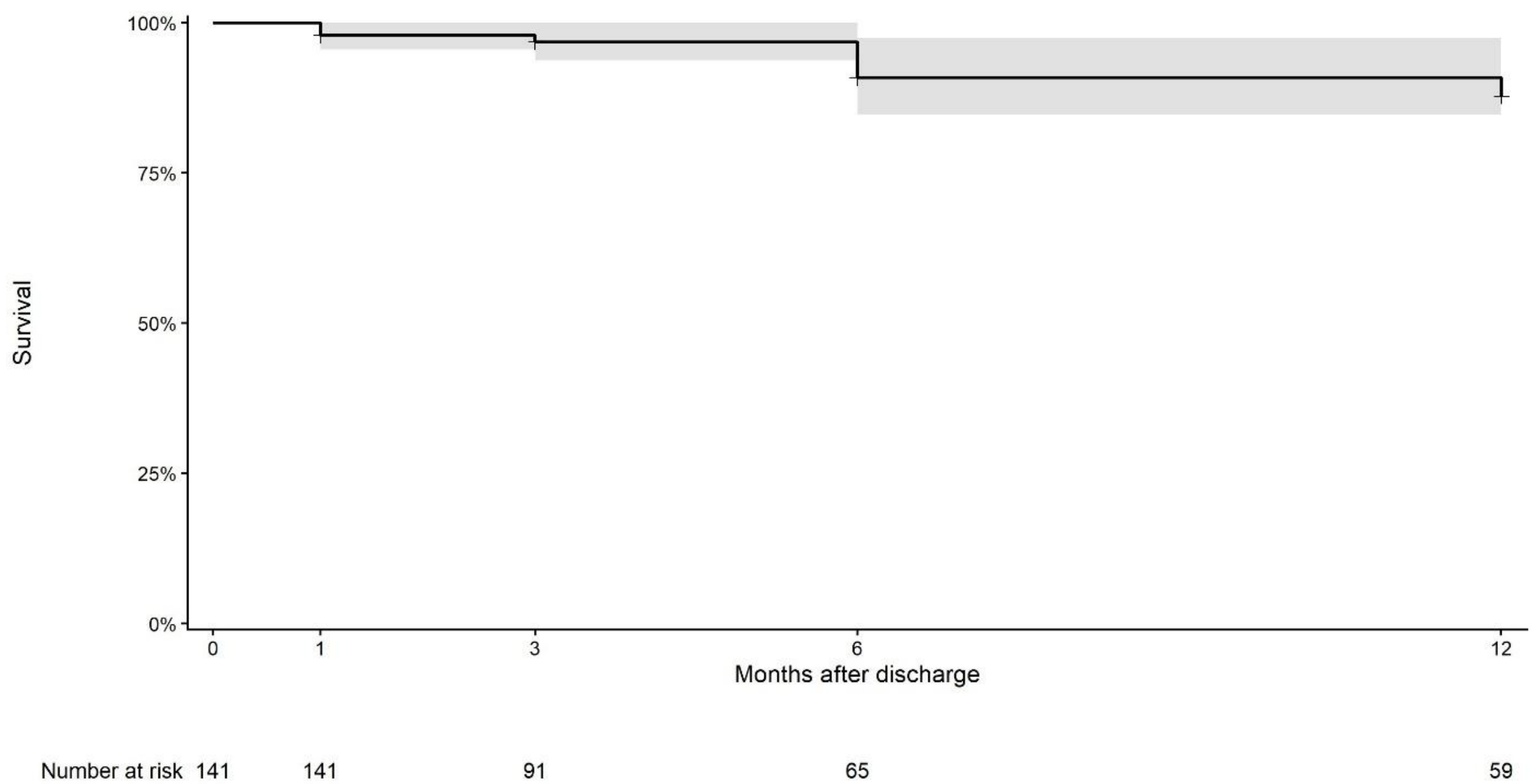

Supplementary Figure S1. Kaplan–Meier estimate of all-cause survival up to 12 months after hospital discharge in patients with viral pneumonia discharged alive (n = 141). The shaded area represents the 95% confidence interval.

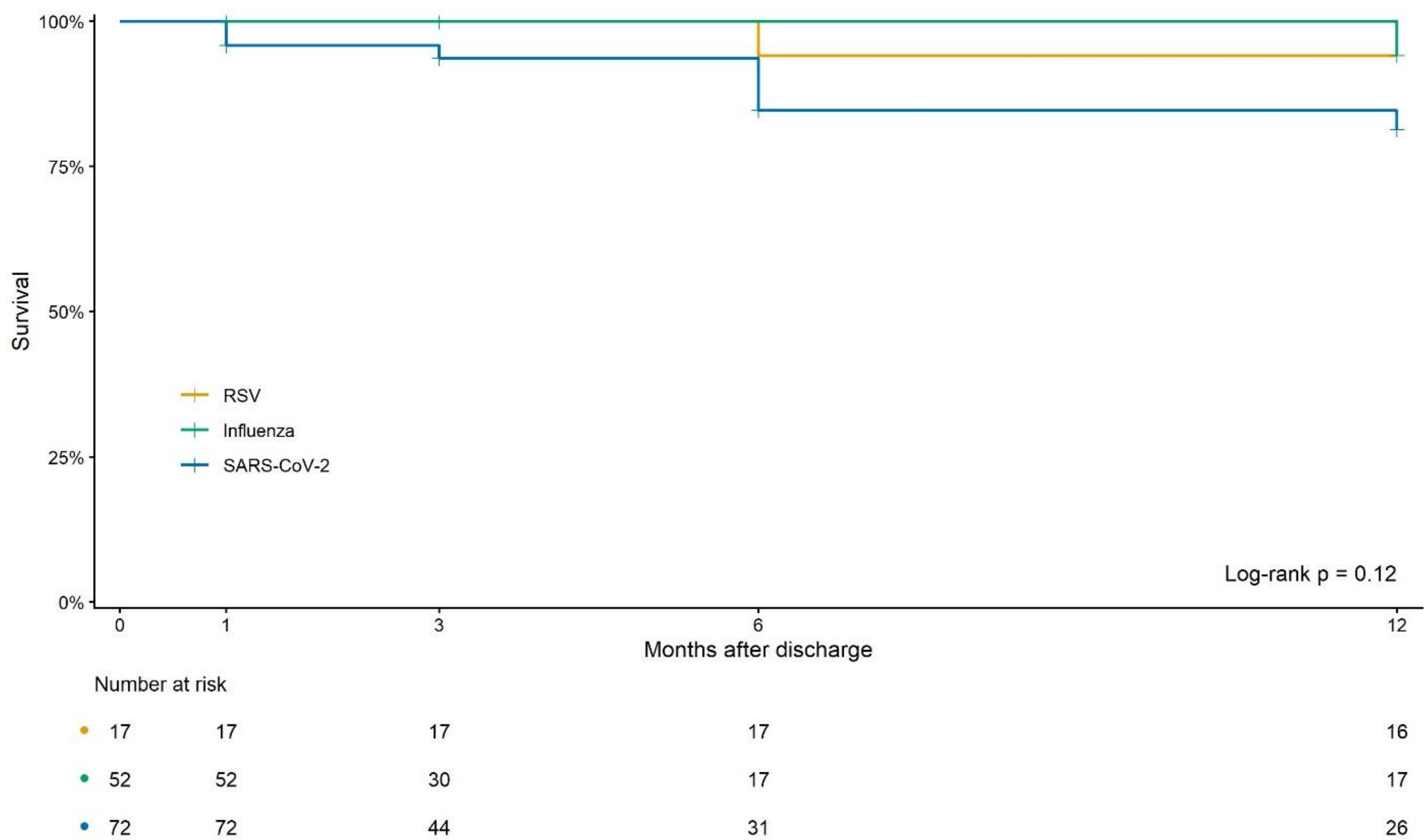

Supplementary Figure S2. Kaplan–Meier estimates of all-cause survival up to 12 months after hospital discharge according to viral etiology (n=141): RSV (n = 17), influenza (n = 52) and SARS-CoV-2 (n = 72). RSV: respiratory syncytial virus.
