# Supplementary material for: Comparative outcomes of influenza, severe acute respiratory syndrome coronavirus 2, and respiratory syncytial virus community-acquired pneumonia: insights from an Italian retrospective cohort study
Source: IJID Reg. 2026 Oct 4;21:101032. doi: 10.1016/j.ijregi.2026.101032 (PMC13757497; doi:10.1016/j.ijregi.2026.101032)
Supplement: Supplementary file 2 [file mmc1.docx]

Supplementary material to:
**Comparative Outcomes of Influenza, SARS-CoV-2, and Respiratory Syncytial Virus Community-Acquired Pneumonia: Insights from an Italian Retrospective Cohort Study**

| **Table of content** | |
| --- | --- |
| **Supplementary Table S1** | **Page nr. 3** |
| **Supplementary Table S2** | **Page nr. 5** |
| **Supplementary Table S3** | **Page nr. 6** |
| **Supplementary Figure S1** | **Page nr. 7** |
| **Supplementary Figure S2** | **Page nr. 8** |
|  |  |
|  |  |
|  |  |
|  |  |
|  |  |
|  |  |
|  |  |

| **Variable** | **Respiratory Syncytial Virus**  **(n=17)** | **Influenza**  **(n=57)** | **SARS-CoV-2 (n=91)** | **P-value** |
| --- | --- | --- | --- | --- |
| **Vital Parameters at admission** | | | | |
| Systolic Blood Pressure, median (IQR) mmHg | 130 (105-172) | 125 (110-143) | 130 (110-146) | 0.648 |
| Diastolic Blood Pressure, median (IQR) mmHg | 75 (68.5- 82) | 74 (65.5-84) | 74 (62-80) | 0.778 |
| Heart Rate, median (IQR) beats/min | 85 (82-104.5) | 96 (82-111.5) | 87.5 (77-100) | 0.064 |
| Respiratory Rate, median (IQR) /min | 18 (18-22) | 18 (18-20.5) | 18 (18-23) | 0.986 |
| Oxygen saturation, median (IQR) % | 95 (94-96) | 95 (92-97) | 95 (90-97) | 0.845 |
| Temperature, median (IQR) °C | 37 (36-38.1) | 37 (36.1-38) | 36.8 (36-38) | 0.951 |
| **Laboratory Tests at admission** | | | | |
| White Blood Cells, median (IQR) /mm³ | 11060 (7130-15070) | 7685 (6175-11680) | 7700 (5735-11175) | 0.101 |
| Hemoglobin, median (IQR) g/dL | 12.8 (11.9-13.5) | 12.3 (10.8-14.4) | 12.5 (11.1-13.8) | 0.747 |
| Platelet Count, median (IQR) ×10³/mm³ | 230 (167.8-252) | 199.5 (136.3-295.1) | 174 (124.5-237) | 0.107 |
| Urea, median (IQR) mg/dL | 42.5 (35.5-83.8) | 51 (37-67) | 52 (35-83) | 0.870 |
| Creatinine, median (IQR) mg/dL | 1.02 (0.8-1.45) | 0.98 (0.7-1.47) | 1.11 (0.85-1.54) | 0.454 |
| AST, median (IQR) U/L | 25 (20-35.5) | 35.5 (27.3-48.8) | 28 (22-47) | 0.061 |
| ALT, median (IQR) U/L | 21 (15-25.5) | 24.5 (16-38) | 20 (14-35) | 0.323 |
| Total Bilirubin, median (IQR) mg/dL | 0.6 (0.5-0.9) | 0.7 (0.5-1) | 0.7 (0.5-0.9) | 0.756 |
| Glucose, median (IQR) mg/dL | 135 (107.5-171) | 116 (102.8- 154) | 120 (102- 160.8) | 0.390 |
| Troponin, median (IQR) ng/L | 19.4 (8.6-32.1) | 24.6 (13.1-43.9) | 23.8 (10.1-81.5) | 0.642 |
| CRP, median (IQR) mg/dL | 14.7 (2.8-17.8) | 10.8 (4.4-17) | 11.4 (4.6-16.5) | 0.997 |
| PCT, median (IQR) ng/mL | 0.36 (0.12-2.93) | 0.36 (0.14-3.27) | 0.36 (0.11-1.12) | 0.806 |
| **Radiology at presentation** | | | | |
| Multilobar infiltrate, N (%) | 13 (76.4) | 43 (75.4) | 63 (69.2) | 0.716 |
| Interstitial pneumonia, N (%) | 1 (5.9) | 23 (40.4) | 61 (67) | <0.001* |
| Pleural effusion, N (%) | 2 (11.8) | 11 (19.3) | 34 (37.4) | 0.018** |
| * p-values: RSV vs. Influenza = 0.008, RSV vs. SARS-CoV2 = <0.001, Influenza vs. SARS-CoV-2= 0.001  ** p-values: RSV vs. Influenza = 0.719, RSV vs. SARS-CoV2 =0.040, Influenza vs. SARS-CoV-2= 0.020 | | | | |
| **Symptoms at admission** | | | | |
| Cough, N (%) | 7 (41.2) | 16 (28.1) | 28 (30.8) | 0.603 |
| Sputum, N (%) | 1 (5.9) | 8 (14) | 9 (9.9) | 0.659 |
| Dyspnea, N (%) | 11 (64.7) | 37 (64.9) | 54 (59.3) | 0.818 |
| Fever, N (%) | 8 (47.1) | 33 (57.9) | 51 (56) | 0.742 |
| Hemoptysis, N (%) | 1 (5.9) | 2 (3.5) | 1 (1.1) | 0.235 |
| Asthenia, N (%) | 6 (35.3) | 34 (59.6) | 29 (31.9) | 0.003* |
| Night Sweats, N (%) | 0 (0) | 1 (1.8) | 0 (0) | 0.448 |
| Weight Loss, N (%) | 0 (0.0) | 6 (10.5) | 4 (4.4) | 0.245 |
| Neurological Symptoms, N (%) | 2 (11.8) | 5 (8.8) | 18 (19.8) | 0.203 |
| Gastrointestinal Symptoms, N (%) | 0 (0.0) | 4 (7) | 5 (5.5) | 0.787 |
| * p-values: RSV vs. Influenza = 0.077, RSV vs. SARS-CoV2 = 0.782, Influenza vs. SARS-CoV-2= <0.001 | | | | |

**Supplementary Table S1.** Vital parameters, laboratory panel, radiology and symptoms at admission in viral pneumonia.

Abbreviations: AST, aspartate aminotransferase; ALT, alanine aminotransferase; CRP, C-reactive protein; PCT, procalcitonin; IQR, interquartile range.

|  | Overall (N = 165) | RSV (N = 17) | Influenza (N = 57) | SARS-CoV-2 (N = 91) | p-value | Missing, n |
| --- | --- | --- | --- | --- | --- | --- |
| **Empirical antibacterial therapy** | | | | | | |
| Any antibacterial agent, N (%) | 129 (78.2) | 15 (88.2) | 49 (86.0) | 65 (71.4) | 0.073 | 0 |
| Monotherapy, N (%) | 63 (38.2) | 5 (29.4) | 21 (36.8) | 37 (40.7) | 0.691 | 0 |
| Combination therapy (≥2 agents) , N (%) | 66 (40.0) | 10 (58.8) | 28 (49.1) | 28 (30.8) | 0.020 | 0 |
| **Antibacterial agents** | | | | | | |
| Ceftriaxone, N (%) | 71 (43.0) | 9 (52.9) | 24 (42.1) | 38 (41.8) | 0.699 | 0 |
| Piperacillin/tazobactam, N (%) | 46 (27.9) | 3 (17.6) | 18 (31.6) | 25 (27.5) | 0.565 | 0 |
| Azithromycin, N (%) | 43 (26.1) | 8 (47.1) | 16 (28.1) | 19 (20.9) | 0.073 | 0 |
| Levofloxacin, N (%) | 19 (11.5) | 1 (5.9) | 13 (22.8) | 5 (5.5) | 0.005 | 0 |
| Vancomycin, N (%) | 5 (3.0) | 1 (5.9) | 2 (3.5) | 2 (2.2) | 0.391 | 0 |
| Meropenem, N (%) | 4 (2.4) | 0 (0.0) | 4 (7.0) | 0 (0.0) | 0.024 | 0 |
| Amoxicillin/clavulanic acid, N (%) | 3 (1.8) | 0 (0.0) | 1 (1.8) | 2 (2.2) | 1.000 | 0 |
| Linezolid, N (%) | 3 (1.8) | 0 (0.0) | 2 (3.5) | 1 (1.1) | 0.682 | 0 |
| Fosfomycin, N (%) | 2 (1.2) | 1 (5.9) | 1 (1.8) | 0 (0.0) | 0.082 | 0 |
| Trimethoprim/sulfamethoxazole, N (%) | 2 (1.2) | 0 (0.0) | 0 (0.0) | 2 (2.2) | 0.617 | 0 |
| Cefiderocol, N (%) | 1 (0.6) | 0 (0.0) | 1 (1.8) | 0 (0.0) | 0.448 | 0 |
| Ceftazidime, N (%) | 1 (0.6) | 1 (5.9) | 0 (0.0) | 0 (0.0) | 0.103 | 0 |
| Ciprofloxacin, N (%) | 1 (0.6) | 1 (5.9) | 0 (0.0) | 0 (0.0) | 0.103 | 0 |
| **Antiviral agents** | | | | | | |
| Remdesivir, N (%) | 42 (25.5) | 0 (0.0) | 0 (0.0) | 42 (46.2) | <0.001 | 0 |
| Oseltamivir, N (%) | 38 (23.0) | 0 (0.0) | 38 (66.7) | 0 (0.0) | <0.001 | 0 |
| Nirmatrelvir/ritonavir, N (%) | 3 (1.8) | 0 (0.0) | 0 (0.0) | 3 (3.3) | 0.485 | 0 |
| Acyclovir, N (%) | 1 (0.6) | 0 (0.0) | 1 (1.8) | 0 (0.0) | 0.448 | 0 |
| **Antifungal agents** | | | | | | |
| Amphotericin B, N (%) | 1 (0.6) | 0 (0.0) | 1 (1.8) | 0 (0.0) | 0.448 | 0 |
| Anidulafungin, N (%) | 1 (0.6) | 0 (0.0) | 1 (1.8) | 0 (0.0) | 0.448 | 0 |
| Voriconazole, N (%) | 1 (0.6) | 0 (0.0) | 0 (0.0) | 1 (1.1) | 1.000 | 0 |

**Supplementary Table S2.** Antimicrobial therapy stated in the first 24 hours from admission to hospital in the study population with diagnosis of viral pneumonia.

|  | aOR | 95% CI | p-value |
| --- | --- | --- | --- |
| Viral etiology (overall) |  |  | 0.40 |
| RSV vs SARS-CoV-2 | 0.31 | 0.002–2.95 | 0.37 |
| Influenza vs SARS-CoV-2 | 0.55 | 0.18–1.51 | 0.26 |
| RSV vs Influenza | 0.57 | 0.004–6.14 | 0.69 |
| PSI class (per 1-class increase) | 3.07 | 1.46–6.99 | **0.002** |

**Supplementary Table S3.** Multivariable logistic regression for in-hospital mortality according to viral etiology, adjusted for PSI class (n = 165). The overall p-value for viral etiology was obtained with a penalized likelihood-ratio test (χ² = 1.82, 2 df). aOR: adjusted odds ratio; CI: confidence interval; PSI: Pneumonia Severity Index; RSV: respiratory syncytial virus.

**Supplementary Figure S1.** Kaplan–Meier estimate of all-cause survival up to 12 months after hospital discharge in patients with viral pneumonia discharged alive (n = 141). The shaded area represents the 95% confidence interval.

**Supplementary Figure S2.** Kaplan–Meier estimates of all-cause survival up to 12 months after hospital discharge according to viral etiology (n=141): RSV (n = 17), influenza (n = 52) and SARS-CoV-2 (n = 72). RSV: respiratory syncytial virus.
